# P. GINGIVALIS–LPS-INDUCED NLRP3 INFLAMMASOME ACTIVATION DRIVES AD PATHOLOGIES

**DOI:** 10.1093/geroni/igad104.2229

**Published:** 2023-12-21

**Authors:** Ambika Verma, Pankaj Patyal, Gohar Azhar, Jeanne Wei

**Affiliations:** University of Arkansas for Medical Sciences, Little Rock, Arkansas, United States; University of Arkansas for Medical Sciences, Little Rock, Arkansas, United States; University of Arkansas for Medical Sciences, Little Rock, Arkansas, United States; University of Arkansas for Medical Sciences, Little Rock, Arkansas, United States

## Abstract

Alzheimer’s disease (AD) is the leading cause of dementia among older adults. AD is characterized by amyloid-β deposition, abnormal hyperphosphorylation of tau leading to the formation of neurofibrillary tangles, and neuroinflammation. Porphyromonas gingivalis, the keystone-pathogen of periodontitis, is being increasingly linked with AD and Alzheimer’s disease-related dementias (ADRD). Poor oral health is common in older populations, and literature suggests that oral health correlates with prevalence of ADRD. NOD-like receptor family pyrin domain containing 3 (NLRP3) inflammasome-mediated IL-1β is significantly involved in periodontal diseases. However, the exact mechanism by which NLRP3 inflammasome is regulated in response to pathogenic bacteria remains unclear. We hypothesized that P. gingivalis-LPS may be responsible for the neuroinflammation via TLR4 activation and its downstream caspase-1, caspase-3 or caspase-4/5/11 dependent cell signaling. Our results suggest that P. gingivalis accelerated the induction of NLRP3. Furthermore, NLRP3/Caspase-1/Caspase-4/5/11 dependent IL-1β production may contribute to the dysregulated neuroinflammatory response in AD pathogenesis.

